# Prevalence of Obesity and Dental Caries in Kindergarten Children During the First Decade of Saudi Vision 2030: A Cross-Sectional Study

**DOI:** 10.3390/children11121531

**Published:** 2024-12-18

**Authors:** Heba M. Elkhodary, Deema J. Farsi, Nada J. Farsi, Logain K. Alattas, Ali B. Alshaikh, Najat M. Farsi

**Affiliations:** 1 Department of Pediatric Dentistry, Faculty of Dentistry, King Abdulaziz University, Jeddah 21589, Saudi Arabia; dfarsi@kau.edu.sa (D.J.F.); nfarsi@kau.edu.sa (N.M.F.); 2 Department of Pedodontics and Oral health, Faculty of Dental Medicine for Girls, Alazhar University, Cairo 11651, Egypt; 3 Department of Dental Public Health, Faculty of Dentistry, King Abdulaziz University, Jeddah 21589, Saudi Arabia; njfarsi@kau.edu.sa; 4 Department of Pediatric Dentistry, Tufts University, Boston, MA 02111, USA; logain.alattas@tufts.edu; 5 Saudi Board of Pediatric Dentistry, Riyadh Elm University, Riyadh 12611, Saudi Arabia; ali.alshaikh2022@student.riyadh.edu.sa

**Keywords:** children, kindergarten, prevalence, obesity, dental caries, BMI, Saudi Vision 2030

## Abstract

Background/Objectives: Obesity and dental caries are significant health issues affecting children worldwide. This study aims to investigate the prevalence of obesity and dental caries among kindergarten children in Saudi Arabia during the early implementation years of the Vision 2030 initiative. Specifically, it examines the obesity rates between public and private kindergartens and assesses the correlation between obesity and caries risk. Methods: We conducted a cross-sectional study involving a stratified sample of 347 kindergarten children in Jeddah, Saudi Arabia, from September 2022 to March 2023, as part of a larger project assessing the obesity and dental caries prevalence in school-aged children. Their body mass index (BMI) was computed after their weight and height were measured. Following an oral examination, the decayed, missing, and filled teeth (dmft) scores were noted. The relationships between dmft and BMI, sex, and school type were studied using non-parametric tests, and its predictors were assessed as well. Results: Our findings indicate that 15.3% of the children were classified as obese based on the BMI measurements, while 9.8% were categorized as overweight. The prevalence of obesity did not show significant differences by school type when classified by BMI. The mean dmft score was 2.8 ± 3.6, with those children in public kindergartens demonstrating significantly higher dmft scores compared to their private counterparts (*p* < 0.001). Notably, there was no observed relationship between obesity and caries activity. Conclusions: Despite the implementation of Saudi Vision 2030, the high prevalence of obesity and dental caries among kindergarten children suggests that the current health initiatives may be insufficient. The lack of a relationship between obesity and caries activity highlights the complexity of these health issues and the need for targeted interventions. To improve the health outcomes, it is recommended to enhance the awareness campaigns regarding oral health and nutrition, increase access to preventive dental care, and integrate nutrition education into kindergarten curricula.

## 1. Introduction

The World Health Organization (WHO) recognizes obesity as a global epidemic and a major concern worldwide [1]. The childhood obesity rates have surged globally, with estimates from 2022 indicating that 37 million children under the age of five were overweight. Initially considered an issue for high-income countries, childhood obesity is now escalating in low- and middle-income nations. Since 2000, the number of overweight children under five in Africa has risen by almost 23%, and nearly half of those affected in 2022 were located in Asia [2].

Body mass index (BMI) is a simple and cost-effective tool used to assess obesity, especially in children and adolescents [3,4]. The preschool years are critical for obesity development as significant metabolic changes occur during this period, posing an increased risk of overweight and obesity later in life [5,6].

In Saudi Arabia, the prevalence of overweight and obesity among kindergarten children has often been underestimated. A study conducted in 2010 reported a prevalence of 18.1% overweight and 19.2% obese among Saudi kindergarten children in Saudi Arabia [7]. More recent research in Jeddah in 2017 found that 10% were overweight and 15% were classified as obese based on BMI [8]. Childhood obesity presents significant health risks, including diabetes, high blood pressure, and elevated cholesterol levels. Studies indicate that around 60% of children who are overweight will continue to be overweight or obese into adulthood [3,9].

Alongside obesity, dental caries is another significant public health issue in Saudi Arabia, worsened by the rising consumption of sugars and processed foods [10]. The rate of dental caries among children is alarmingly high, estimated at around 80% for primary dentition, affecting both genders [11]. The relationship between poor dental health and obesity is multifactorial, influenced by lifestyle, dietary habits, and socioeconomic status [12,13]. Some studies suggest a possible connection between dental caries and overweight/obesity, indicating that higher body weight may be associated with increased caries experience among children [14,15]. However, the findings remain inconsistent, with other studies reporting no correlation [12,16,17]. Systematic reviews have also yielded mixed results, highlighting the need for more research to understand this connection [18,19].

In response to pressing health challenges, Saudi Arabia launched “Vision 2030” in 2016, announced by Crown Prince Mohammed Bin Salman [20], emphasizing healthcare improvements through initiatives such as establishing nutrition clinics, the RASHAKA program, the “Prevent Tooth Caries” initiative, and physical activity programs like Sports For All [21,22,23,24,25]. Preventive measures targeting overweight and dental health among kindergartens could facilitate a multidisciplinary approach to improve the overall child health in the country. Thus, the current study aims to investigate the obesity and dental caries prevalence among kindergarten children in Saudi Arabia during the first years of the Vision 2030 program’s implementation. Specifically, the objectives are the following: (1) examining the obesity and dental caries prevalence among this age group; (2) comparing the obesity rates between public and private kindergarten children; and (3) determining the risk of caries in overweight, obese, or underweight kindergarten children compared to their normal-weight peers. By establishing baseline data on these interrelated pediatric health issues, this research can help to inform policymakers and healthcare providers in developing targeted interventions that align with the goals of “Vision 2030.”

## 2. Methods

### 2.1. Ethics Statements

This study received approval from the Ethical Committee of the Faculty of Dentistry at King Abdulaziz University (KAUFD) under approval number 064-06-20. Parental consent was secured for all participants.

### 2.2. Design and Participants

The current cross-sectional study was conducted among kindergarten children in Jeddah, Saudi Arabia, between September 2022 and March 2023. This research is a phase of a comprehensive study examining obesity and dental caries prevalence among school children across three different age groups. The findings for adolescents have already been published [26], while research for elementary school children is still in progress. Currently, we are focusing on kindergarten children. About 25% of kindergarten children in Saudi Arabia are overweight or obese [8]. For this study, we calculated a sample size of 300 students, equally divided between boys and girls, with an 85% power and a 5% alpha, anticipating a 30% outcome frequency and a 3% confidence limit. Children aged three to six with primary teeth, and whose parents had returned signed consent forms, were eligible for inclusion in the study. The Saudi Ministry of Education in Jeddah reported that there were 32,091 children enrolled in kindergartens. Kindergartens were chosen at random from a current list that was categorized by financing source (private or public) and district (West, East, South, or North). Each strata had one kindergarten chosen, for a total of eight kindergartens. To achieve the necessary sample size, one class was chosen at random from each kindergarten. There were two phases to the recruitment of children. Children were provided a questionnaire to gather demographic information and a parental consent form during the initial appointment. After returning the signed parental consent form, eligible children completed a clinical assessment during the second visit.

### 2.3. Anthropometric Measurements

Two calibrated investigators conducted anthropometric measurements using a non-elastic measuring tape. Height was determined as the distance from the top of the head to the floor. For accurate weight assessment, children removed their shoes, jackets, and accessories and were weighed on an electronic scale. For analysis, two readings were taken for every measurement, and they were averaged. Body mass index was computed by dividing height (m) squared by weight (kg). The intra-examiner and inter-examiner reliability for the anthropometric measurements were assessed using the kappa statistic, with scores of 0.86 and 0.83, respectively.

### 2.4. Dental Examination

The oral examinations were performed by two calibrated investigators using disposable instruments, including cotton rolls, gauze, a dental probe with rounded end, and a sterilized intraoral flat-surface mirror. The dental examination was conducted using a torchlight, following basic infection control measures in a designated room within the kindergarten, specifically set up for this purpose. The children were positioned in front of the examiner on a school chair during the procedure. The dmft index, which stands for decayed, missing, and filled teeth, was used to record caries experience in accordance with WHO standards. The intra-examiner and inter-examiner reliability for the dental examinations were assessed, with kappa scores of 0.90 and 0.88, respectively. Temporary restorations, restorations with secondary caries, and teeth with enamel surface structural losses were all marked as decayed. White spot lesions and sealants were considered sound. A confidential letter of referral to KAUFD was issued to the child’s parent after examination, outlining the child’s oral health and treatment requirements.

### 2.5. Statistical Analysis

Categorical variables, such as gender and school type, were presented as counts and percentages, while continuous variables, such as biometric measures and age, were presented via means and standard deviations. BMI classification in our study was based on Saudi guidelines [27], utilizing the following thresholds: obese (95th percentile and above), overweight (85th to 94th percentiles), normal weight (5th to 84th percentiles), and underweight (below the 5th percentile) [28]. The percentage of obesity among participants was assessed, accompanied by 95% confidence interval (CI). The prevalence of obesity was then stratified by gender and school type to assess their association with obesity. The distribution of dmft was assessed in relation to gender, school type, and BMI categories. To compare the means between two groups, two-sample *t*-tests were utilized. For comparisons involving more than two groups, one-way analysis of variance (ANOVA) was employed. The normality of continuous variables was assessed prior to conducting parametric tests by conducting the Shapiro–Wilk test, visual inspection of histograms, and Q–Q plots. Various count data models, including Poisson, negative binomial, zero-inflated Poisson, and zero-inflated negative binomial models, were used to identify predictors of dmft. The Akaike Information Criterion (AIC) was used to select the best-fitting model, with the zero-inflated negative binomial model showing the lowest AIC values, indicating the best fit. This selection was supported by the observation that more than 40% of the dmft values were zero, and an overdispersion in dmft was noted. All statistical tests were two-tailed, with a *p*-value of 0.05 or less considered statistically significant. All analyses were conducted using Stata/IC 12.1 (StataCorp LP, College Station, TX, USA).

## 3. Results

Of the 441 children invited to participate in the study, 347 were included, resulting in a response rate of 78.7%. As shown in Table 1, the children involved in the study had an average age of 4.7 ± 0.8 years, with 50.4% being boys. The majority of the children attended private kindergartens (74.1%). Regarding parent education, 68.3% of the fathers and 58.5% of the mothers had university degrees, respectively. Only a small proportion of the parents had postgraduate education degrees, 10.1% of the fathers and 4.0% of the mothers. The mean weight of the participating children was 19.5 ± 5.5, and the mean height was 111.4 ± 9.5, while the mean dmft was 2.8 ± 3.6.

Table 2 demonstrates the prevalence of obesity among the participants. Of the children, 71.5% (95% CI: 66.7–76.2%) were of normal weight, while 3% (95% CI: 1.5–5.4%) were underweight. Overweight and obese children comprised 9.8% (95% CI: 6.7–12.9%) and 15.3% (95% CI: 11.5–19.1%) of the participants, respectively.

The prevalence of obesity, when analyzed by gender and school type, did not show statistically significant associations, as presented in Table 3.

Table 4 illustrates the distribution of the dmft scores, categorized by gender, school type, and BMI. No significant differences were observed in the dmft scores between boys and girls. The underweight children seemed to have higher dmft scores than the normal-weight, overweight and obese children; however, this did not reach statistical significance. The children in public kindergartens had higher dmft scores than those children in private kindergartens (*p* < 0.001).

The predictors of higher dmft scores are presented in Table 5. The multivariate analysis, which accounted for confounding variables, identified that those children who attended private kindergartens had lower dmft scores when compared with the children who went to public kindergartens. Also, the underweight children had higher dmft scores when compared with the normal-weight children. However, fathers’ education was not significantly associated with dmft scores.

## 4. Discussion

This cross-sectional study investigated the obesity prevalence among private and public kindergarten children of ages from 3 to 6 years. Additionally, it assessed the association between obesity and dental caries in primary teeth. The kindergarten children were generally in the normal-weight group, with a mean BMI of 15.5 ± 2.9. When it comes to prevalence, obesity almost equally affects boys and girls. Similarly, the children attending public kindergartens had comparable caries activity for boys and girls but considerably higher activity for caries in comparison to those attending private kindergartens. There was no observed relationship between obesity and caries activity. The study found that 25.1% of the kindergarten children were overweight or obese based on BMI, highlighting an important public health concern. This finding is consistent with a similar study conducted eight years ago among a comparable population in Jeddah [8]. However, this percentage is higher than the 20.7% prevalence found in a study conducted in the Eastern Province among preschoolers aged 2 to 4 years [7]. Another recent study analyzed data from various regions in Saudi Arabia between 2016 and 2021, focusing on children and teenagers of ages between 2 and 19 years. It was observed that the children of ages between 2 and 6 years had the highest obesity rate of 12.3% according to the report [29]. Conversely, Tehran had a lower prevalence, with overweight and obesity rates of 2.2% and 5.0%, respectively, among 3–5-year-old children [30]. The variations in the obesity rates in these studies could be because of different cultural and dietary practices in various regions, along with variations in how overweight and obesity are measured.

The current study found no significant gender differences in the BMI categories among the kindergarten-aged children in Saudi Arabia, which aligns with earlier research [15]. This suggests that, during the kindergarten years, environmental and developmental factors likely impact boys and girls similarly, influenced by factors like the physical environment [31] and parental eating habits [32]. However, a previous study in Jeddah found that girls had slightly higher rates of both overweight/obesity and underweight compared to boys, although these differences were not statistically significant [8]. A possible explanation may be that subtle gender-based variations can start emerging at this young age in certain regional contexts, potentially influenced by socio-cultural norms, household dynamics, or other environmental factors. Overall, boys and girls in Saudi Arabia appear to have similar growth patterns, but localized studies might uncover important gender-based differences that warrant further exploration.

Dental caries is very prevalent among children in Saudi, with an overall prevalence rate of 65.6%. The prevalence in primary teeth is even higher, at 72.1%, with a mean dmft of 3.93 (±3.60) [33]. In contrast, the mean dmft observed in our study population was lower, at 2.6 (3.8). This finding is lower compared to the mean dmft of 5.0 reported by Al Agili in 2013 in a systematic review on primary dentition [11]. Although this pattern of a lower mean dmft aligns with previous national studies, it is important to note that our research focused specifically on kindergartens in the city of Jeddah, which may limit the generalizability of these results to the wider national population. In the current study, the caries distribution was similar between the girls and boys, contrasting with a previous study that indicated slightly higher caries activity in boys, although this difference was not statistically significant [15]. Remarkably, those children in public kindergartens exhibited significantly higher caries experience than those in private kindergartens. This aligns with the findings from a study in Greece, which reported a low caries prevalence among children attending private daycare centers [34]. This disparity may be attributed to the socioeconomic backgrounds of the children as those in public kindergartens are more likely to come from lower-income families that often face barriers to accessing adequate oral health care. In contrast, children in private kindergartens typically have parents with greater financial means, enabling them to access better dental care and preventive services [35]. A multivariate analysis revealed that the children in private kindergartens had lower dmft scores than their public school counterparts, likely due to those families with higher socioeconomic status having better resources for oral hygiene and greater awareness of oral health [36].

In this study, we discovered no association between obesity/overweight and dental caries. This is consistent with prior research, which also did not find an association between dental caries and BMI among kindergarten children [12,17]. It is possible that children who are obese or overweight may eat more fatty foods compared to their underweight or healthy-weight peers yet might not necessarily consume more sugary foods. This could explain the lack of a direct link between obesity and dental caries observed in this study. However, there have been studies that have found strong associations between caries and obesity [3,14,15,37]. The studies by Bafti et al. [38] and Elkodary et al. [8] among preschoolers even found negative associations. Systematic reviews, exploring the link between caries and BMI categories among children, have yielded mixed results [18,39]. A systematic review by Chen et al. in 2018 found no significant differences in the caries rates between abnormal-weight groups and normal-weight groups for both primary and permanent teeth [40]. These findings imply that there is a complex link between childhood obesity and dental caries that cannot be fully explained by carbohydrate intake [41]. Several other factors might influence this relationship, including the age of the children, with older children demonstrating a stronger association [14]. Socioeconomic status serves as a crucial determinant, with children from disadvantaged backgrounds more likely to be obese or overweight and exhibiting higher rates of early childhood caries [40,42]. Interestingly, in our study, the underweight children displayed the highest mean decay score compared to their normal-weight, overweight, and obese counterparts, although this difference did not reach statistical significance. This observation is consistent with the findings from an Indian study that reported a similar trend among underweight children [43], but it is essential to note that our results do not establish a definitive relationship. The recurring pattern across studies indicates that underweight status may be a risk factor for poorer dental health, potentially influenced by factors such as nutrition, oral hygiene, impaired immune function, and socioeconomic conditions. While our study found that the underweight children had higher dmft scores compared to their normal-weight peers, the lack of statistical significance suggests that these variations may not be conclusive and could be affected by confounding variables.

The study had several strengths. The researchers employed a careful methodological approach utilizing standardized study instruments to ensure data accuracy and reliability. This study was part of a broader project investigating obesity and dental caries prevalence among school children, aligning with the 2030 vision. Moreover, the researchers integrated insights from local studies to provide accurate context, involving a diverse sample of children attending public and private kindergartens across all the city districts. However, despite these strengths, the study faced several limitations. The cross-sectional design restricted the data collection to a single time point, posing challenges in establishing causal relationships. Additionally, as the study was confined to one city, the outcomes may not be fully indicative of the entire country. The omission of prevalent risk factor data such as dietary habits and physical activity could have impacted the perceived correlation between obesity and dental caries. Future research should consider implementing surveys to study children’s dietary and oral hygiene behaviors, use of fluoride, and access to care. By incorporating these variables, researchers can gain full qualitative insights, providing a more comprehensive understanding of the influences on caries prevalence. Kindergarten children in Saudi Arabia, as a vulnerable group, require targeted interventions for both obesity and dental caries [44]. Addressing dietary habits [10,45], parental influence [18], and healthcare access [46,47] is imperative for establishing lifelong healthy behaviors and averting long-term consequences. Engaging parents, caregivers, and healthcare providers is crucial for addressing these interconnected health issues during the formative years. Healthcare providers play a pivotal role in educating parents and instigating behavior changes to combat obesity in young populations. Implementing public health programs and early interventions align with Saudi Vision 2030′s emphasis on promoting healthy lifestyles and preventive care, particularly for young children. Effective targeted efforts for kindergarten children can significantly enhance the overall public health and well-being.

## 5. Conclusions

This study revealed that, while the majority of the kindergarten children were within the normal weight range, the prevalence of obesity and dental caries among these children remains high. The obesity prevalence was similar among boys and girls, and the caries activity was notably higher in the public kindergarten children compared to those in private settings. Importantly, no association was found between obesity and dental caries. These findings indicate that, despite the implementation of Saudi Vision 2030, the high rates of obesity and dental caries among the kindergarten children suggest that the current health initiatives may need to be strengthened. To improve the outcomes, it is essential to enhance awareness campaigns focusing on oral health and nutrition, empower families with resources, and increase access to preventive dental care. Regular screenings in kindergartens can facilitate early identification of dental issues, while integrating nutrition education into curricula will promote healthier eating habits. Collaboration with parents and caregivers is crucial for fostering healthy lifestyle choices. The continuous evaluation of the existing programs and the introduction of new evidence-based interventions are vital for effectively addressing these public health challenges.

## Figures and Tables

**Table 1:** Sociodemographic and biometric characteristics of the participants (n = 347).

Variable	N (%) *
Age, mean (SD)	4.7 (0.8)
Gender	
Boys	175 (50.4)
Girls	172 (49.6)
School type	
Public	90 (25.9)
Private	257 (74.1)
Health	
No	31 (8.9)
Yes	316 (91.1)
Father education	
High school or less	75 (21.6)
University	237 (68.3)
Postgraduate	35 (10.1)
Mother education	
High school or less	130 (37.5)
University	203 (58.5)
Postgraduate	14 (4.0)
Income (SAR)	
>4000	19 (5.5)
4000–3800	301 (86.7)
<3800	27 (7.8)
Weight (kg), mean (SD)	19.5 (5.5)
Height (cm), mean (SD)	111.4 (9.5)
BMI, mean (SD)	15.5 (2.9)
dmft, mean (SD)	2.8 (3.6)

BMI: body mass index; dmft: decayed, missed, filled teeth index; SAR 1 = USD 0.27. * Number (percentage) unless indicated otherwise.

**Table 2:** Prevalence of obesity.

Obesity Category	Proportion (95% CI *)
Underweight	3 (1.5–5.4)
Normal	71.5 (66.7–76.2)
Overweight	9.8 (6.7–12.9)
Obese	15.3 (11.5–19.1)

* 95% CI: 95% confidence interval.

**Table 3:** Prevalence of obesity stratified by gender and school type.

Variable	Gender		*p*-Value	School Type		*p*-Value
	Boys	Girls		Public	Private	
Normal	125 (71.4)	123 (71.5)		68 (75.6)	180 (70.0)	
Underweight	7 (4.0)	5 (2.9)	0.846 ^	0 (0)	12 (4.7)	0.084 *
Overweight/ obese	43 (24.6)	44 (25.6)		22 (24.4)	65 (25.3)	

^ Chi-square test; * Fisher’s exact test.

**Table 4:** Distribution of dmft by gender, school type, and BMI.

Variable	dmft		*p*-Value
	Mean (SD)	Median (IQR)	
Gender			
Boys	2.8 (3.6)	1 (0, 5)	0.983 *
Girls	2.8 (3.7)	1 (0, 5)	
School type			
Public	4.4 (4.2)	3 (1, 8)	<0.001 *
Private	2.3 (3.3)	0 (0, 4)	
BMI			
Normal	2.8 (3.5)	1 (0, 5)	0.065 ^
Underweight	5.3 (5.4)	3 (0, 10)	
Overweight/obese	2.7 (3.8)	1 (1, 4)	

* Two-sample *t*-test; ^ one-way analysis of variance (ANOVA).

**Table 5:** Predictors of dental caries (dmft) among the participants (zero-inflated negative binomial model).

Variable	N	Univariate Regression Coefficient (95% CI *)	Multivariate Regression Coefficient (95% CI *)
Gender			
Boys	175	1.0	---
Girls	172	0.03 (−0.2–0.2)	
School type			
Public	90	1.0	1.0
Private	257	−0.2 (−0.4–−0.01)	−0.2 (−0.4–−0.01)
Nationality			
Saudi	297	1.0	---
Non-Saudi	50	0.02 (−0.3–0.3)	
Health			
No	31	1.0	---
Yes	316	0.2 (−0.1–0.5)	
Father education			
High school or less	75	1.0	1.0
University	237	−0.1 (−0.3–0.1)	−0.03 (−0.3–0.2)
Postgraduate	35	−0.4 (−0.8–−0.04)	−0.3 (−0.6–0.1)
Mother education			
High school or less	130	1.0	---
University	203	−0.1 (−0.3–0.1)	
Postgraduate	14	0.1 (−0.4– 0.6)	
Income (SAR)			
>4000	19	1.0	---
4000–3800	301	−0.1 (−0.5–0.2)	
<3800	27	−0.4 (−1.0–0.1)	
BMI			
Underweight/Normal	260	1.0	---
Overweight/obese	87	−0.03 (−0.3–0.2)	
BMI			
Normal	248	1.0	1.0
Underweight	12	0.5 (0.01–0.9)	0.5 (0.1–1.0)
Overweight/obese	87	−0.0 (−0.2–0.2)	−0.0 (−0.2–0.2)

* 95% CI: 95% confidence interval.

## Data Availability

All data generated for this study belong to the current research team and are available from the corresponding authors upon reasonable request to protect the data privacy and restrict unauthorized use.
